# Rapid and profound treatment response of difficult-to-treat rheumatoid arthritis to CD19 CAR T-cell therapy

**DOI:** 10.1016/j.ero.2025.08.008

**Published:** 2025-09-18

**Authors:** Fredrik N. Albach, Ioanna Minopoulou, Artur Wilhelm, Arnd Kleyer, Edgar Wiebe, Anja Fleischmann, Klaus Engel, Mareike Frick, Frederik Damm, Julia Gogolok, Sebastian Serve, Benjamin Locher, Dominic Borie, Elpida Phithak, Lukas Hinkelmann, Sarah Ohrndorf, Vincent Casteleyn, Robert Biesen, Arne Sattler, Thomas Dörner, Norman M. Drzeniek, Tobias Alexander, Jan Zernicke, Nadine Unterwalder, Kamran Movassaghi, Marie Luise Hütter-Krönke, Eva Schrezenmeier, Adrian Schreiber, Christian Furth, Georg Schett, Udo Schneider, Lars Bullinger, Gerhard Krönke, Olaf Penack, David Simon

**Affiliations:** 1 Department of Rheumatology and Clinical Immunology, Charité - Universitätsmedizin Berlin, Berlin, Germany; 2 German Rheumatism Research Centre (DRFZ) Berlin, a Leibnitz Institute, Berlin, Germany; 3 Siemens Healthineers, Erlangen, Germany; 4 Department of Hematology, Oncology and Tumor Immunology, Charité - Universitätsmedizin Berlin, Campus Virchow Klinikum, Berlin, Germany; 5 Kyverna Therapeutics, Emeryville, CA, USA; 6 Fachbereich Allergiediagnostik, Labor Berlin – Charité Vivantes GmBH, Berlin, Germany; 7 Department of Hematology, Oncology and Cancer Immunology, Charité - Universitätsmedizin Berlin, Campus Steglitz, Berlin, Germany; 8 Department of Nephrology and Medical Intensive Care, Charité-Universitätsmedizin, Berlin, Germany; 9 Department of Nuclear Medicine, Charité - Universitätsmedizin Berlin, Berlin, Germany; 10 Department of Internal Medicine 3, Friedrich-Alexander-Universität (FAU) Erlangen-Nürnberg and Universitätsklinikum Erlangen, Erlangen, Germany; 11 Deutsches Zentrum für Immuntherapie (DZI), FAU Erlangen-Nürnberg and Universitätsklinikum Erlangen, Erlangen, Germany; 12 Experimental and Clinical Research Center, Charité - Universitätsmedizin Berlin and Max Delbrück Center for Molecular Medicine in the Helmholtz Association, Berlin, Germany

New treatments, along with the implementation of a “treat-to-target” strategy, have significantly improved the prognosis of patients with rheumatoid arthritis (RA). However, a subset of patients, classified as having difficult-to-treat RA (D2T RA), still fail to respond adequately to currently available treatment options, resulting in poor disease outcomes and reduced quality of life [1]. Development of novel therapeutic strategies in this subset of patients is crucial. Initial reports have suggested that CD19 chimeric antigen receptor (CAR) T-cell therapy may provide a therapeutic benefit in RA. However, in these reports, (1) RA overlapped with other autoimmune diseases [2,3], (2) significant side effects including higher-grade cytokine release syndrome (CRS) and immune effector cell–associated neurotoxicity syndrome (ICANS) were observed [4], or (3) complex vectors that combined CD19 targeting with interleukin 6/tumor necrosis factor alpha (IL6/TNFα) neutralisation were used [5]. Thus, the efficacy and safety of standard CD19 CAR T-cell therapy in D2T RA remain uncertain.

Herein, we present a 70-year-old man with RA (disease duration of 20 years) with erosive D2T RA and positivity for anticitrullinated protein antibodies (ACPAs) and rheumatoid factor (RF) who was treated with CD19 CAR T-cell therapy (KYV-101, Kyverna Therapeutics). KYV-101 is a fully human, autologous anti-CD19 CAR T-cell therapy with CD28 costimulation, designed for potency and tolerability. At no point in his medical history was a state of stable disease remission achieved despite (1) many years of glucocorticoid therapy (2) treatment with various conventional disease-modifying antirheumatic drugs (DMARDs) (methotrexate [2004-2008; 2022-2023], leflunomide [2008], and sulfasalazine [since 2023]), and (3) multiple targeted therapies (adalimumab [2004], etanercept [2007-2011], certolizumab [2011-2012], tocilizumab [2012-2019], tofacitinib [2019-2020], baricitinib [2020-2021], abatacept [2021-2022], rituximab [2022], sarilumab [2023], and golimumab [since 2023]), with cumulative DMARD prescription costs of approx >300.000 euros. All agents, except methotrexate (discontinued in 2023 owing to gastrointestinal intolerance) and leflunomide (terminated owing to exanthema in 2008), were discontinued owing to insufficient efficacy.

Owing to the uncontrolled and persistently high clinical disease activity (disease activity score [DAS] 28-erythrocyte sedimentation rate [ESR], 5.46) together with serological and imaging findings (Fig, A,C; Supplementary Fig S1 and Supplementary Video 1) and in light of multiple prior treatment failures, severely impaired quality of life, and the explicit wish of the patient, CD19 CAR T-cell therapy was initiated through shared decision making. No relevant contraindications such as uncontrolled infection, severe comorbidities, or organ dysfunction were present.

**Figure:**
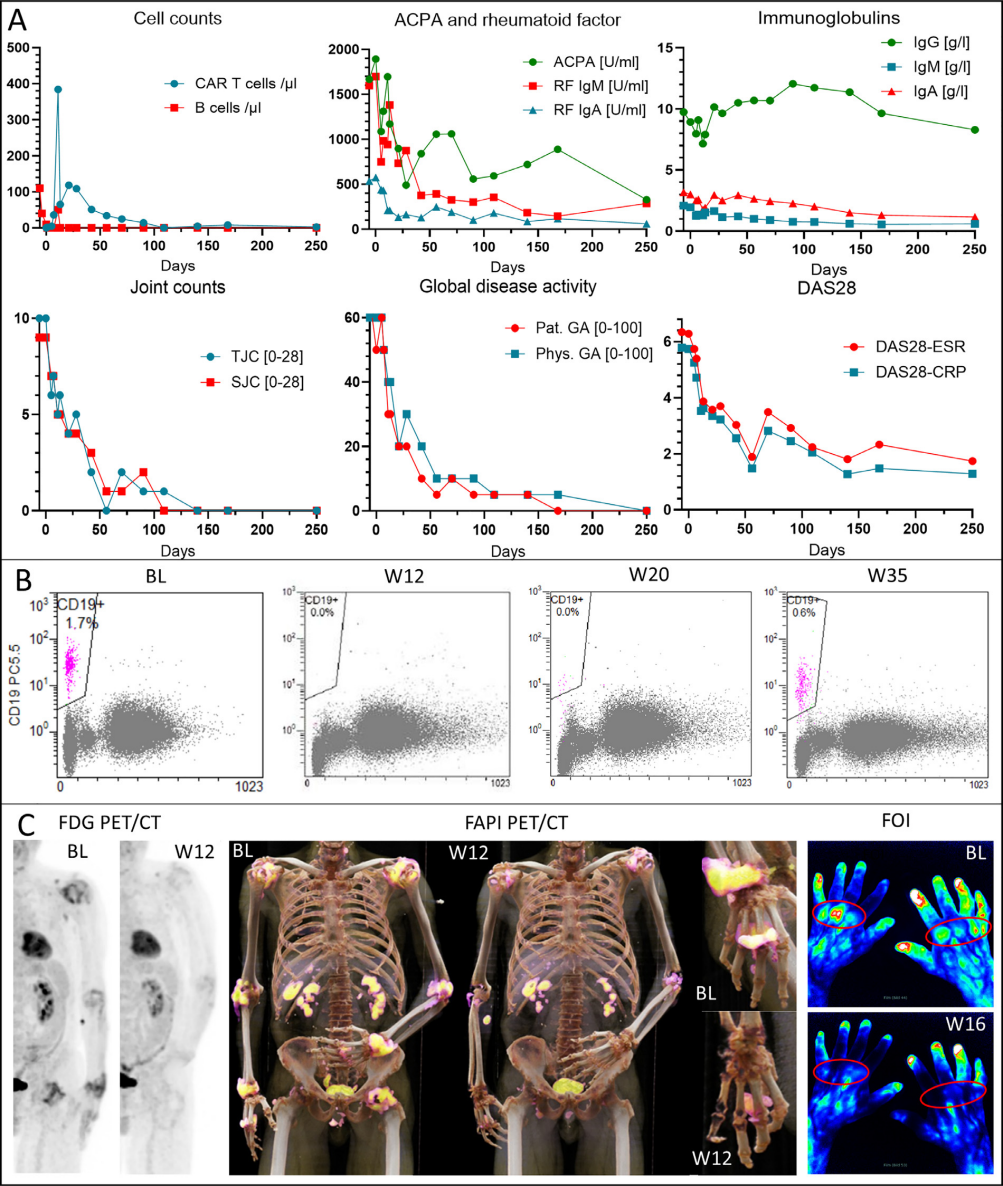
Immunological, clinical, and imaging response to CD19 chimeric antigen receptor (CAR) T-cell therapy. (A) Graphs show expansion of CAR T cells and depletion of B cells, decline in anticitrullinated protein antibodies (ACPAs) and rheumatoid factor (RF) immunoglobulin M (IgM)/immunoglobulin A (IgA), as well as reductions in tender and swollen joint counts, patient and physician global activity, and disease activity scores (DAS)-28-CRP/ESR after CD19 CAR T-cell therapy. (B) Flow cytometry analyses of peripheral blood showed complete depletion at week (W) 12, followed by reconstitution of CD19+ B cells at W35 after CAR T-cell therapy. (C) Maximum intensity projections of 18-fluorodeoxyglucose (FDG) positron emission tomography (PET)/computed tomography (CT) imaging and cinematic rendering of Gallium 68 (^68^Ga)-labelled fibroblast activation protein inhibitor (FAPI) PET/CT imaging at baseline (BL) and 12 weeks after CD19 CAR T-cell therapy showed a significant decline in joint inflammation (in shoulders, elbows, wrists, metacarpophalangeal [MCP] joints, and left hip joint), and fluorescence optical imaging (FOI) showed early and strong enhancement within MCP joints of both hands (MCP II–IV of the left and MCP II–V of the right hand, most notably at MCP III of the left hand, red circles) at BL and a normal result without signs of MCP joint inflammation16 weeks after treatment. TJC: tender joint count; SJC: swollen joint count; Pat.: patient; Phys.: physician; GA: global activity; ESR: erythrocyte sedimentation rate; CRP: C-reactive protein.

Immunosuppressive treatment with golimumab and sulfasalazine was stopped before leukapheresis, transiently resumed during the manufacturing period, and then withdrawn 4 and 2 weeks prior to initiating lymphodepletion, whereas prednisone (5 mg/d) was discontinued 1 week prior to treatment. The patient received 1.0 × 10^8^ CD19 CAR T cells (day 0) following lymphodepletion with 30 mg/m^2^ fludarabine and 300 mg/m^2^ cyclophosphamide daily for 3 days (days −5 to −3).

After CD19 CAR T-cell infusion, the patient experienced grade II CRS, which fully resolved within 7 days following treatment with tocilizumab and dexamethasone (cumulative dose, 68 mg). No ICANS, infections, or bone marrow toxicity were observed up to the latest follow-up (month 8, week 35).

CD19 CAR T cells rapidly expanded after infusion and peaked after 10 days (384.22 CAR T/µL; 31.51% of total CD3+ cells), before gradually declining over the subsequent 12 weeks. Peripheral CD19+ B cell counts rapidly declined, demonstrating sustained B cell depletion (Fig, A). Flow cytometry analysis confirmed complete peripheral CD19+ B cell depletion at week 12 and demonstrated B cell reconstitution 35 weeks after treatment (Fig, B). ACPA and RF levels markedly declined from baseline values through month 8 after treatment (ACPA, 1892 vs 329 U/mL [reference range, <20 U/mL]; RF immunoglobulin M [IgM], 1697 vs 288 U/mL [reference range, <20 U/mL]; RF immunoglobulin A [IgA], 576 vs 60 U/mL [reference range, <20 U/mL]). Interestingly, ACPAs showed a decline at month 8 despite B cell reconstitution. Immunoglobulins remained within normal limits (Fig, A), and vaccination titres against measles, rubella, and varicella remained almost unchanged (Supplementary Table S1).

Despite the complete withdrawal of immunosuppressive treatment, a rapid and sustained drug-free amelioration of the disease activity reaching remission was achieved (DAS28-ESR 1.74 at month 8), which was accompanied by a substantial reduction in inflammation detected by whole-body high-resolution metabolic imaging (Fig, C, Supplementary Video 1), musculoskeletal ultrasound (Supplementary Fig S1), and fluorescence optical imaging (Fig, C) as well as a markedly reduced fatigue (FACIT score improved from 31 before therapy to 46 at month 8).

In summary, these findings demonstrate clinical efficacy of CD19 CAR T-cell therapy in D2T RA with a favourable safety profile. Consistent with previous reports, this case further substantiates the potential of CD19 CAR T-cell therapy in D2T RA. The present case is distinguished by the advanced age of the patient, the longest follow-up reported to date, and the application of a comprehensive whole-body imaging approach, thereby reinforcing the robustness of this emerging therapeutic paradigm [[2], [3], [4], [5]].

As in haematologic malignancies, CAR T-cell therapy in autoimmune diseases faces certain limitations (eg, manufacturing complexity) and uncertainties regarding long-term safety. In RA, the durability of immune reset, as observed in other autoimmune diseases, also remains to be determined [6].

Ongoing clinical trials, such as the COMPARE trial (NCT06475495), a randomised controlled phase I/II trial in D2T RA comparing CD19 CAR T-cell therapy with rituximab, are expected to provide important insights into long-term efficacy and safety. These results may ultimately clarify whether CAR T-cell therapy can initiate a paradigm shift in RA by enabling a durable reset of autoimmunity and sustained drug-free remission.

## Editor disclosure

The peer review process did not involve Editorial Board Members Arnd Kleyer and David Simon, and the editorial decision-making was led by editors who were not involved in the creation of this manuscript.

## CRediT authorship contribution statement

**Fredrik N. Albach:** Writing – review & editing, Writing – original draft, Visualization, Validation, Project administration, Methodology, Investigation, Formal analysis, Data curation, Conceptualization. **Ioanna Minopoulou:** Writing – review & editing, Writing – original draft, Methodology, Investigation, Formal analysis, Data curation, Conceptualization. **Artur Wilhelm:** Investigation, Conceptualization. **Arnd Kleyer:** Writing – review & editing, Supervision, Resources, Methodology, Funding acquisition, Conceptualization. **Edgar Wiebe:** Writing – review & editing, Investigation. **Anja Fleischmann:** Investigation. **Klaus Engel:** Visualization. **Mareike Frick:** Supervision. **Frederik Damm:** Supervision. **Julia Gogolok:** Investigation. **Sebastian Serve:** Investigation. **Benjamin Locher:** Investigation. **Dominic Borie:** Conceptualization. **Elpida Phithak:** Writing – review & editing, Investigation. **Lukas Hinkelmann:** Writing – review & editing. **Sarah Ohrndorf:** Visualization, Methodology, Investigation. **Vincent Casteleyn:** Writing – review & editing. **Robert Biesen:** Writing – review & editing, Supervision. **Arne Sattler:** Methodology, Data curation. **Thomas Dörner:** Writing – review & editing, Supervision. **Norman M. Drzeniek:** Writing – review & editing, Methodology. **Tobias Alexander:** Writing – review & editing, Validation, Supervision. **Jan Zernicke:** Resources, Project administration. **Nadine Unterwalder:** Visualization, Investigation. **Kamran Movassaghi:** Resources, Investigation. **Marie Luise Hütter-Krönke:** Visualization, Validation, Supervision, Data curation. **Eva Schrezenmeier:** Writing – review & editing, Validation. **Adrian Schreiber:** Validation. **Christian Furth:** Visualization, Investigation, Data curation. **Georg Schett:** Writing – review & editing, Validation, Supervision. **Udo Schneider:** Supervision, Investigation, Conceptualization. **Lars Bullinger:** Data curation. **Gerhard Krönke:** Writing – review & editing, Validation, Supervision, Resources, Methodology, Funding acquisition, Data curation, Conceptualization. **Olaf Penack:** Writing – review & editing, Validation, Supervision, Methodology, Investigation, Data curation, Conceptualization. **David Simon:** Writing – review & editing, Validation, Supervision, Resources, Project administration, Methodology, Investigation, Funding acquisition, Data curation, Conceptualization.

## Competing interests

IM received speaker honoraria from AbbVie and Novartis. DS has served on scientific advisory boards for AbbVie, Bristol-Myers Squibb, Gilead Sciences, Janssen-Cilag, Lilly, Novartis, and UCB and received speaker honoraria from AbbVie, Alfasigma, Bristol-Myers Squibb, Janssen-Cilag, Kyverna, Lilly, Novartis, and UCB. AK has served on scientific advisory boards for AbbVie, Bristol-Myers Squibb, Gilead Sciences, Janssen-Cilag, Lilly, Novartis, and UCB and received speaker honoraria from AbbVie, Alfasigma, Bristol-Myers Squibb, Janssen-Cilag, Lilly, Novartis, and UCB. TD received honoraria for scientific advice from AbelZeta, BMS, J&J, Novartis, and Roche/Genentech. TA received honoraria from AbbVie, Amgen, and Johnson & Johnson. DB is an employee and shareholder of Kyverna Therapeutics. OP has received honoraria or travel support from Alexion, Gilead, Jazz, MSD, Neovii, Novartis, Pfizer, and Therakos; has received research support from Incyte and Priothera, and is a member of the advisory boards of Apogepha, Alexion, Equillium Bio, Jazz, Gilead, Novartis, MSD, Omeros, Orca Bio, Priothera, Sanofi, Shionogi, and SOBI.

## Appendix Supplementary materials

Image, application 1

Image, video 2 dummy alt text

## Footnotes

Supplementary material associated with this article can be found in the online version at doi:10.1016/j.ero.2025.08.008.
