# Supplementary material for: Rapid and profound treatment response of difficult-to-treat rheumatoid arthritis to CD19 CAR T-cell therapy
Source: EULAR Rheumatol Open. 2025 Sep 18;2(2):100053. doi: 10.1016/j.ero.2025.08.008 (PMC13425153; doi:10.1016/j.ero.2025.08.008)
Supplement: Supplementary file 1 [file mmc1.docx]

**Supplement**


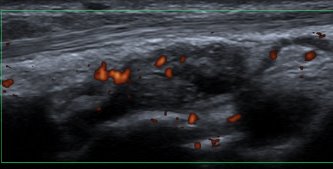


BL

**
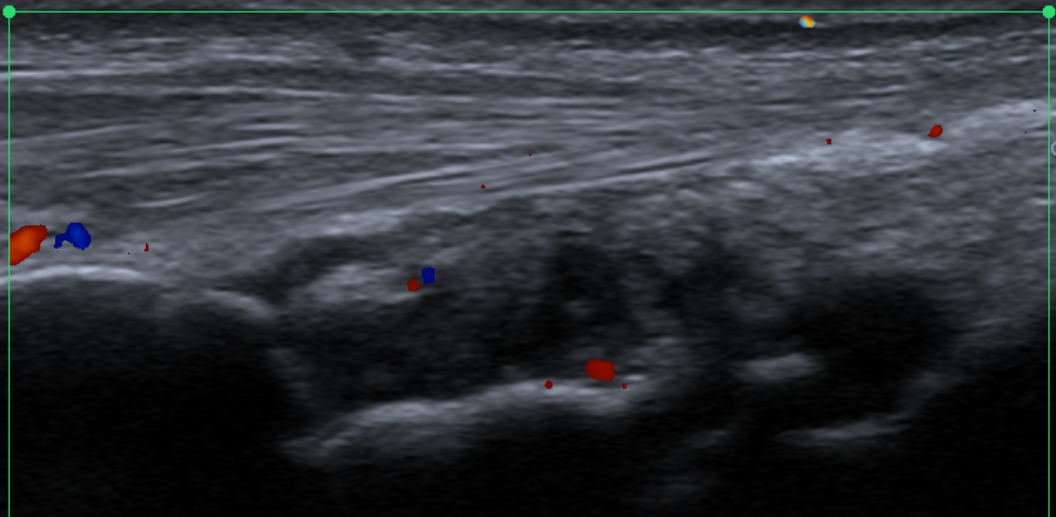

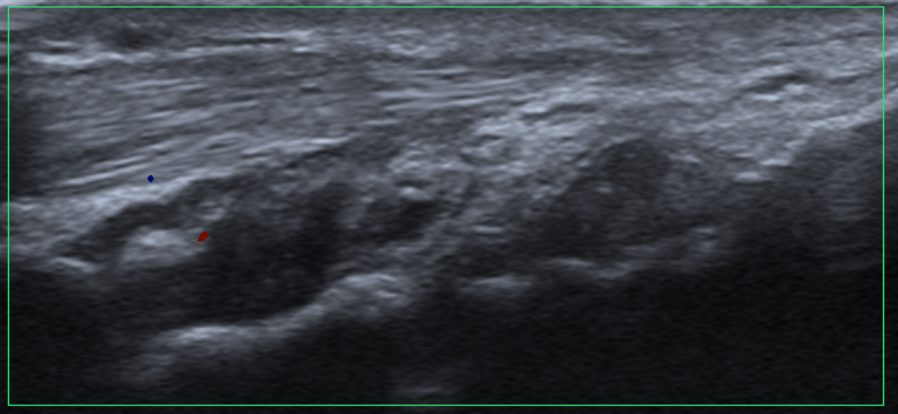
**

W4

W6

**Supplementary Figure 1.** Musculoskeletal ultrasound examination with significant reduction of Power Doppler signal (right radiocarpal joint, dorsomedian view) from grade 2 before CD19 CAR-T cell therapy (baseline = BL) to 0 after 6 weeks (W6)

| **Days after CD19 CAR T-cell therapy**  W16  BL | **Baseline** | **28** | **42** | **56** | **70** | **90** | **109** | **140** | **168** |
| --- | --- | --- | --- | --- | --- | --- | --- | --- | --- |
| **Anti-measles IgG** | pos. | pos. | pos. | pos. | pos. | pos. | pos. | pos. | pos. |
| **Anti-mumps IgG** | pos. | pos. | pos. | pos. | pos. | pos. | pos. | pos. | pos. |
| **Anti-rubella IgG [IU/ml]** | 37.10 | 39.40 | 49.90 | 60.70 | 62.60 | 65.20 | 64.30 | 55.1 | 59.0 |
| **Anti-varicella-zoster IgG [mIU/ml]** | 989 | 1046 | 1676 | 1617 | 1918 | 1597 | 1703 | 1859 | 1688 |
| **Anti-tetanustoxoid IgG [IU/ml]** | 0.44 | 5.69 | 5.41 | 5.22 | 2.25 | 1.41 | 3.14 | 1.58 | 0.85 |
| **Anti-EBV-VCA IgG [U/ml]** | 633 | >750 | 712 | 619 | >750 | 724 | >750 | 744 | >750 |
| **Anti-EBV-EBNA1 IgG [U/ml]** | 49.2 | 17.70 | 14.70 | 20.90 | 14.80 | 13.70 | 15.00 | 18.4 | 16.1 |
| **Anti-cytomegalovirus IgG [AE/ml]** | >250 | >250 | >250 | >250 | >250 | >250 | >250 | >250 | >250 |

**Supplementary Table 1.** Vaccination and post-infection titers before and after CD19 CAR T-cell therapy show sustained levels of protective titers

**Supplementary Video.** Cinematic rendering of FAPI PET/CT shows significant amelioration of joint inflammation 12 weeks after CD19 CAR T-cell therapy (in shoulders, elbows, wrists, metacarpophalangeal joints, left hip joint).
